# Hemostatic bioactivity of novel *Pollen Typhae Carbonisata*-derived carbon quantum dots

**DOI:** 10.1186/s12951-017-0296-z

**Published:** 2017-09-05

**Authors:** Xin Yan, Yan Zhao, Juan Luo, Wei Xiong, Xiaoman Liu, Jinjun Cheng, Yongzhi Wang, Meiling Zhang, Huihua Qu

**Affiliations:** 1 School of Chinese Materia Medica, Beijing University of Chinese Medicine, Beijing, 100029 China; 2 School of Basic Medical Sciences, Beijing University of Chinese Medicine, Beijing, 100029 China; 3 Center of Scientific Experiment, Beijing University of Chinese Medicine, 11 Beisanhuandong Road, Chaoyang District, Beijing, 100029 China

**Keywords:** *Pollen Typhae Carbonisata*, Carbon quantum dots, Hemostasis, Charcoal drug, Drug discovery

## Abstract

**Background:**

*Pollen Typhae Carbonisata* (PTC) is a type of calcined herb drug that has been used as a hemostatic medicine to promote hemostasis for thousands of years. In this study, we discovered and separated novel water-soluble carbon quantum dots (CQDs, named PTC-CQDs) from aqueous extracts of PTC. These PTC-CDs were characterized using transmission electron microscopy (TEM) and high-resolution TEM, as well as Fourier transform infrared, ultraviolet–visible, and fluorescence spectroscopy. Then, we assessed the anti-hemorrhagic effects and related hemostatic mechanisms of the obtained PTC-CQDs.

**Results:**

The PTC-CQDs separated from PTC are spherical, monodisperse, and have a narrow size distribution between 2 and 8 nm. In the pharmacology experiment, remarkable anti-hemorrhage effects of PTC-CQDs were revealed. Additionally, the rats showed a profound decrease in activated partial thromboplastin time and increase in fibrinogen and PLT after PTC-CQDs treatment.

**Conclusions:**

These results indicated the explicit hemostasis effect of PTC-CQDs, which not only provided a new idea for the material research of PTC, but have also provided new insights into potential biomedical and healthcare applications of CQDs in the field of haemorrhage control and laid a solid foundation for future drug discovery.

## Background

*Pollen Typhae* (PT), named Pu Huang in Chinese, is a traditional folk herbal medicine widely used in China, Korea, and Japan. The charcoal processed product of PT, *Pollen Typhae Carbonisata* (PTC), has been widely used to treat hemorrhagic conditions for many years in Traditional Chinese Medicine (TCM), was recorded in the *Compendium of Materia Medica* during the Ming Dynasty (1578 A.D., in China) at the earliest, and acknowledged in the 2015 Pharmacopoeia of the People’s Republic of China (PPRC) as a hemostatic drug.

Supported by consecutive clinical evidence and literature recordation, the haemostatic effect of PTC is clear. Modern medical researches have indicated that the hemostatic function of some drugs could be produced after charcoal processing [1–4]. However, for years, numerous efforts have been expended to elucidate the material basis of PTC and other charcoal drugs from the perspective of small molecule active compound, but the results have been unsatisfactory. This challenging predicament compelled us to turn our attention on a new generated substances after charcoal processing, carbon quantum dots (CQDs), but which are absent in the original crude herb.

Nanomaterials, such as fluorescent semiconductor and graphene quantum dots (QDs), have attracted the interest of many medical researchers for in vivo molecular and cellular imaging [5] and drug delivery [6]. Recently, their self-bioactivities and pharmacological effects have also gained attention, but still in the early stages. It was reported that graphene QDs could enhanced the osteogenic differentiation of mesenchymal stem cells [7] and had antagonistic capacity against the adverse effects of ethanol [8]. In another study, the CdTe QDs were identified as potent inhibitors of the nuclear factor (NF)-kB pathway with both in vitro and in vivo experiments, which provide evidence establishing the potential for the use of QDs in the development of novel anti-cancer, anti-viral, and anti-inflammatory approaches [9].

As another important type of nanomaterial emerging recently, CQDs have superior properties compared with those of other nanomaterials in terms of high aqueous solubility, low toxicity, and good biocompatibility [10]. However, the application of CDs in the biomedical domain was mainly focused on drug delivery [11, 12], bio-imaging [13, 14], and optical imaging and sensing [15, 16]. The intrinsic bioactivity and potential pharmacological effects of CQDs have not attracted adequate attention, and has become a ‘blind spot’ in research.

In this paper, we report the discovery and purification of a new type of CQDs from the water extract of PTC, which was named PTC-CQDs. We characterized the PTC-CQDs using transmission electron microscopy (TEM) and high-resolution TEM (HRTEM), as well as fluorescence, ultraviolet–visible (UV–vis), and Fourier transform infrared (FTIR) spectroscopy. Then, we assessed the anti-hemorrhagic effects and related hemostatic mechanisms of the obtained PTC-CQDs.

## Methods

### Chemicals

PT was purchased from the local pharmacy and dried in rotating-drum, forced-air drying oven for 2 h at 50 °C. Hemocoagulase (HC) for injection was purchased from Jinzhou Ahon Pharmaceutical Co., Ltd., (Liaoning, China). Dialysis membrane of 1000 Da molecular weight cutoff (MWCO) was purchased from Beijing Ruida Henghui Technology Development Co., Ltd., (Beijing, China). Pentobarbital sodium and other analytical-grade chemical reagents were obtained from Sinopharm Chemical Reagents Beijing (Beijing, China). All of the experiments were performed using deionized water.

### Animals

Male Kunming mice (weighing 30.0 ± 1.0 g) and Sprague–Dawley (SD) rats (weighing 190 ± 10 g) were purchased from the Laboratory Animal Center, Si Beifu with a Laboratory Animal Certificate of Conformity. Animals were maintained under the following conditions: temperature, 24.0 ± 1.0 °C; relative humidity, 55–65%; and a 12-h light/dark cycle with ad libitum access to food and water.

### Preparation of PTC-CQDs

The PTC was prepared using a modified pyrolysis method in a muffle furnace (TL0612, Beijing Zhong Ke Aobo Technology Co., Ltd., China). First, the dried PT was placed into a crucible, which was covered with aluminum foil paper, before closing the lid to form a tight seal. The PT was calcined using a muffle furnace for 1 h at 350 °C to produce PTC. After cooling to room temperature, the PTC was finally ground into a fine powder.

**Fig. 1:**
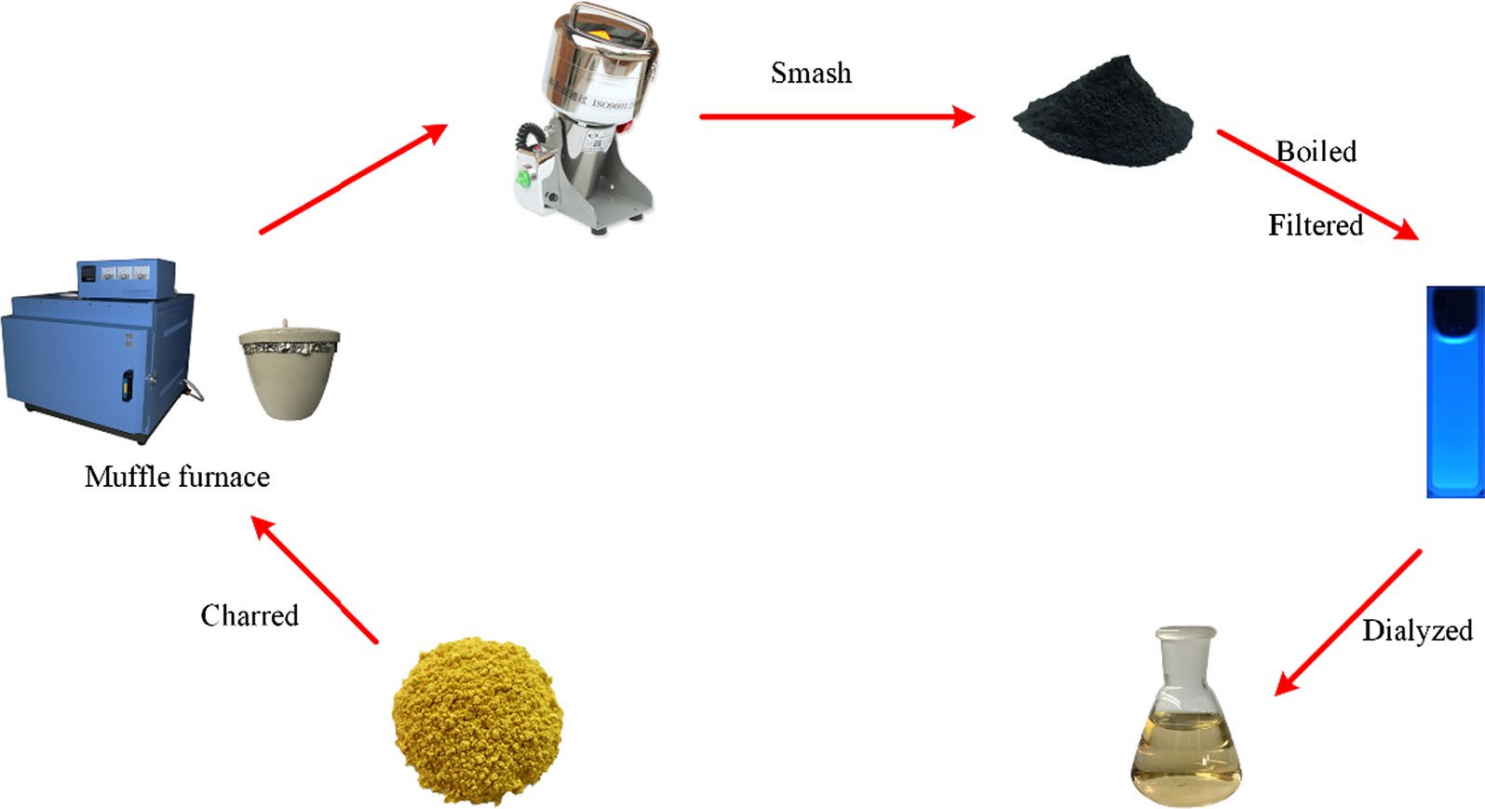
Flow diagram of the *Pollen Typhae Carbonisata*-carbon quantum dots (PTC-CQDs) preparation process

To separate and obtain the PTC-CQDs, the fine PTC powder was soaked in 200 mL deionized water (DW) and boiled thrice for 1 h each. Then, the resulting solution was filtered to remove residue, concentrated, and dialyzed against DW for 96 h using a 1000 Da MWCO dialysis membrane to purify the PTC-CQDs. The entire preparation process for the PTC-CQDs is shown in Fig. 1.

### Characterization of PTC-CQDs

A comparative analysis between PT and the obtained PTC-CQDs solution was performed by high-performance liquid chromatography (HPLC) according to the literature [17] with some modification to evaluate their components as well as the component changes with calcination. Aqueous solution of PTC-CQDs and the methanol extract of PT were initially prepared. Chromatographic separation was conducted using an Agilent series 1260 HPLC instrument (Agilent, Waldbronn, Germany) comprising a quaternary pump, a diode-array detector, an autosampler, and a column compartment. A Reliash-C_18_ column (250 mm × 4.6 mm, Orochem, Illinois, USA) packed with 5 mm octadecyl-bonded silica (C_18_) was used for separation of the CDs. The mobile phases A and B were 0.1% methane acid and acetonitrile respectively, all of which were filtered through 0.22 µm cellulose acetate membrane filters (Jin Teng, Tianjin, China) prior to use. A gradient elution program was applied at a flow rate of 1 mL/min as follows: 0–40 min, 87–80% A, 13–20% B; 40–50 min, 80–75% A, 20–25% B; and 50–70 min, 75–50% A, 25–50% B. The injection volume was 10.0 μL and the column temperature was maintained at 25 °C. The sample signal was monitored by absorbance at 248 nm.

The morphology, size, and microstructure of the resultant PTC-CQDs were characterized by TEM (Tecnai G2 20, FEI Company., USA), operating at an accelerating voltage of 200 kV. The structural details and the atomic lattice fringes of PTC-CQDs were examined by HRTEM (JEN-1230, Japan Electron Optics Laboratory, Japan).

The spectral properties were studied using UV–vis spectroscopy (CECIL, Cambridge, UK) and fluorescence spectroscopy (F-4500, Tokyo, Japan) in a standard quartz cuvette. In addition, the FTIR (Thermo, California, USA) spectra was recorded to identify the organic functional groups in the PTC-CQDs within a spectral window of 400–4000 cm^−1^.

### Hemostasis studies of PTC-CQDs

The mouse tail amputation and liver scratch models were established according to previous methods [18, 19] with modifications. Briefly, 80 male Kunming mice (weighing 30.0 ± 1.0 g, 40 of each model) were divided into the following groups and were injected subcutaneously as indicated: control (normal saline [NS]); HC (0.1 KU/mL); and high-, medium-, and low-dose PTC-CQDs (8, 4, and 2 mg/kg, respectively). For the experiment, the animals were anesthetized with 4% pentobarbital sodium via intraperitoneal injection (0.07 mL/30 g). In the tail amputation model, the mouse tails were transected using a sterile scalpel at the point where the tail diameter was 1.10–1.14 mm (approximately 10 mm from the tip). Then, each tail was immediately placed on filter paper. In the liver scratch model, the liver injury was established by scratching the left lateral lobe with a 2-mL syringe, causing the liver to bleed, and then the incision was overlaid with filter paper. The bleeding was monitored at 30 s intervals until hemostasis was achieved. The hemostatic endpoint was defined as the maintenance of hemostasis for 30 min [4], and the time was recorded until the hemorrhage ceased. All of the mice were euthanized via cervical dislocation under anesthesia at the end of each experiment.

### Measurement of coagulation parameters

A total of 40 SD rats (weighing 190.0 ± 10.0 g) were randomly divided into the following five groups (n = 8/group) and subcutaneously injected as indicated: control (NS); HC (0.1 KU/mL); and high-, medium-, and low-dose PTC-CQDs (8, 4, and 2 mg/kg, respectively). Two hours after the subcutaneous injections, blood samples were withdrawn from the aorta abdominalis and then placed in plastic tubes with 3.2% sodium citrate (citrate/blood: 1/9, v/v) for at least 15 min. Then, the samples were centrifuged at 3000 rpm for 15 min to obtain plasma. Routinely, coagulation parameters, including the values of activated partial thromboplastin time (APTT), prothrombin time (PT), thrombin time (TT), and fibrinogen (FIB), were measured using an automatic coagulation analyzer [20]. Meanwhile, another 60 μL whole blood was collected for the determination of the platelet count (PLT).

### Statistical analysis

Statistical analysis was performed using the statistical package for the social sciences (SPSS, version 17.0). The non-normally distributed data were expressed as the median (quartile range). The within group differences were assessed using a non-parametric test, while the Wilcoxon signed-rank test was used to compare two groups.

The normally distributed data with equal variances were expressed as the mean ± standard deviation. Multiple comparisons were performed using a one-way analysis of variance (ANOVA) followed by the least significant difference (LSD) test. A P < 0.05 was considered statistically significant for the analyses.

## Results

### Characterization of PTC-CQDs

**Fig. 2:**
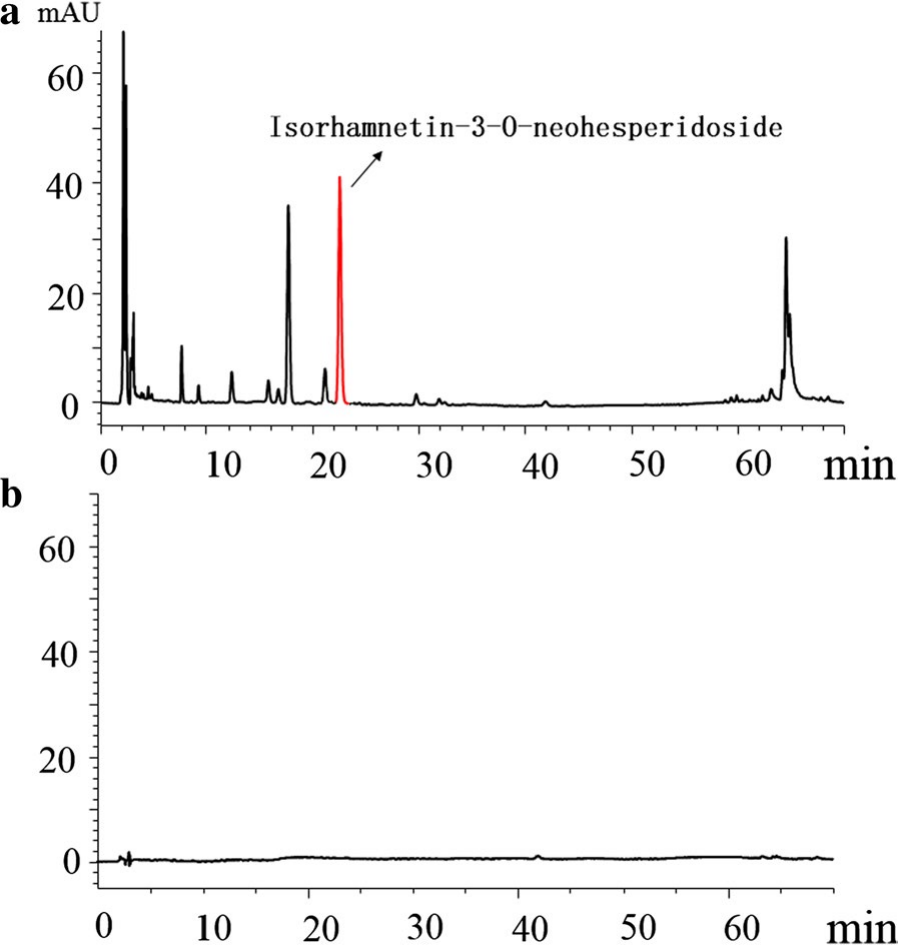
HPLC profile of *Pollen Typhae* (PT) extract (**a**) and *Pollen Typhae Carbonisata* carbon quantum dots (PTC-CQDs) solution (**b**)

Figure 2a shows the HPLC fingerprint of PT Extract. A series of peaks for small-molecule compounds were observed, and the specific peak of index component, isorhamnetin-3-*O*-neohesperidoside, was indicated. Figure 2b shows the HPLC profile for PTC-CQDs under the same conditions. It is obvious that the small molecular components that existed in PT had disappeared in the PTC-CQDs solution.

**Fig. 3:**
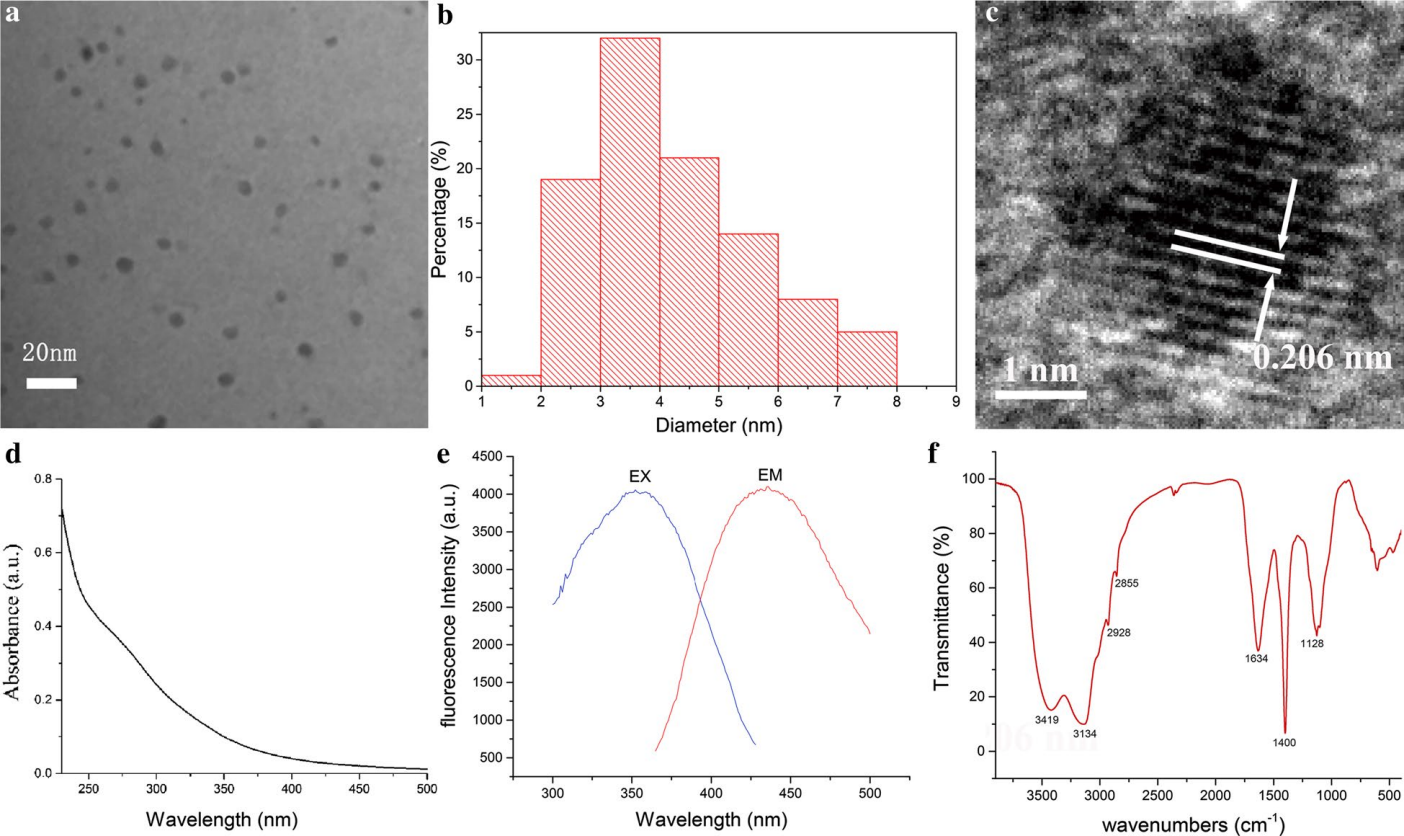
**a** Transmission electron microscopy (TEM) images of *Pollen Typhae Carbonisata* carbon quantum dots (PTC-CQDs) displaying ultra-small particles. **b** Histogram depicting particle size distribution. **c** High-resolution TEM (HRTEM) image of PTC-CQDs. **d** Ultraviolet–visible (UV–vis) and **e** fluorescence spectra and spectrum. **f** FTIR spectrum

The PTC-CQDs extracted from the PTC solution were characterized in the present study. As shown in Fig. 3a, the TEM image of the PTC-CQDs clearly reveal that the CQDs are spherical, monodisperse, and have a narrow size distribution between 2 and 8 nm, with a maximum population at 3-5 nm and mean size of 4.2 ± 1.4 (Fig. 3b). Furthermore, the HRTEM shows that the CQDs had a lattice spacing of 0.206 nm (Fig. 3c).

The UV–vis absorption was also analyzed to determine the optical properties of the PTC-CQDs. The UV–vis spectrum of the aqueous PTC-CQDs solution exhibited a broad absorption pattern that dropped from 200 nm, but showed no obvious peak (Fig. 3d). Furthermore, the fluorescence spectra showed maximum emission at 442 nm and maximum excitation at 352 nm (Fig. 3e).

To gain a better insight into the organic functional groups on the surface of the PTC-CQDs, we further analyzed the CQDs using FTIR, and the spectra of purified PTC-CQDs (Fig. 3f) show characteristic peaks at 3419, 3134, 2928, 2855, 1634, 1400, and 1128 cm^−1^. The presence of O–H groups is indicated by the peak at 3419 cm^−1^. The weak absorption signals at 2928 and 2855 cm^−1^ were attributed to –C–H stretching, which may occur because of the association of methyl or methylene groups with the aliphatic hydrocarbons present in the PTC-CQDs. Moreover, the absorption peak at 1634 cm^−1^ in the PTC-CQDs is the characteristic absorption of C=O. In addition, the peak at 1400 cm^−1^ in the PTC-CQDs spectrum is related to the –C=N– band and the weak C–O stretching band occurs at ~1128 cm^−1^ (alkoxy) [21, 22].

### Hemostasis studies of PTC-CQDs

**Fig. 4:**
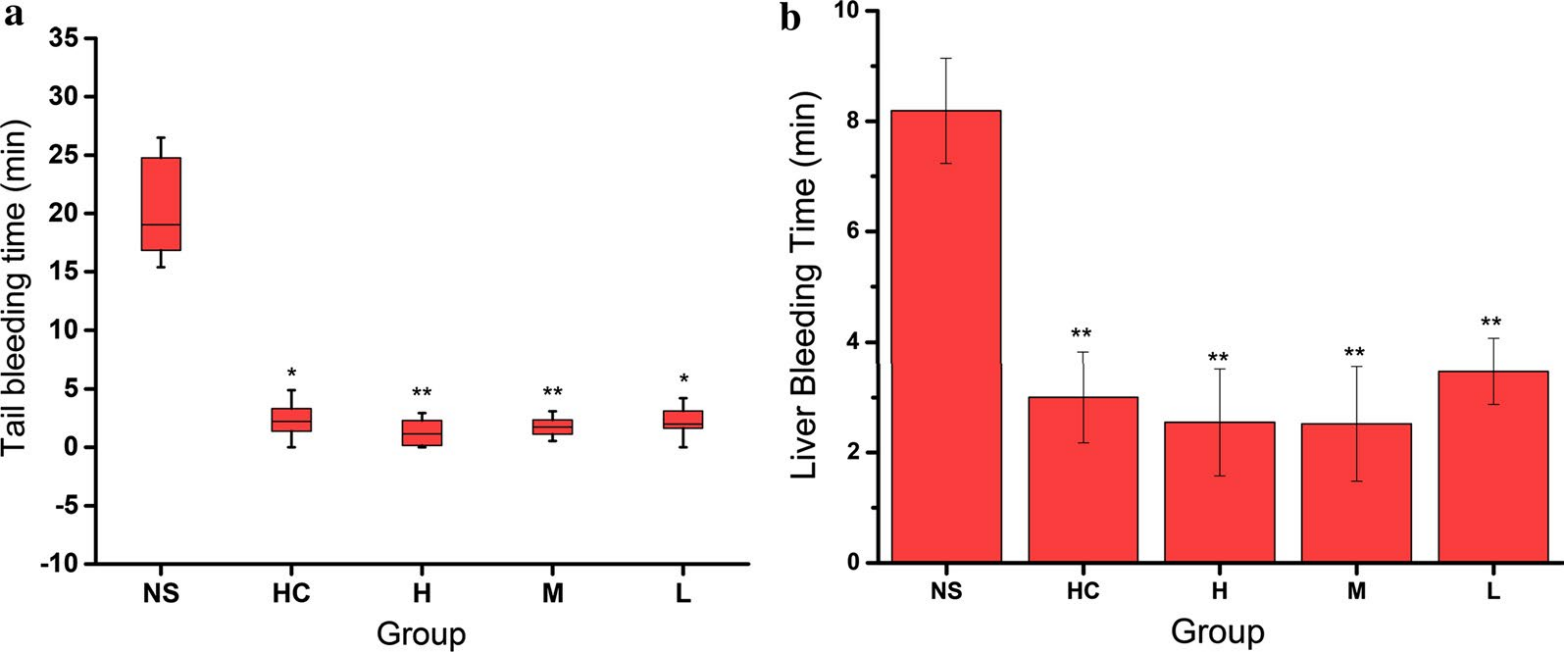
Time to hemostasis in **a** Kunming mouse tail amputation (*box* and *whisker plot*, n = 8) and **b** liver scratch (mean ± standard deviation, n = 8) models treated with normal saline (NS), hemocoagulase (HC), and high (H), medium (M), and low (L) doses of *Pollen Typhae Carbonisata* carbon dots (PTC-CQDs). **P < 0.01 and *P < 0.05 compared with control group

The hemostatic effect of PTC-CQDs was explored using mouse tail amputation and liver scratch models. A significant decrease in the bleeding time was observed after PTC-CQDs treatment in both injury models (Fig. 4).

The tail bleeding time decreased in the PTC-CQDs-treated groups at high [1.2 (2.5) min], middle [1.7 (1.4) min], and low [1.99 (1.5) min] doses compared with that in the control group [19.0 (8.2) min, P < 0.01].

In the liver scratch model, high-, medium-, and low-dose PTC-CQDs, and HC treatment significantly decreased (P < 0.01) the liver bleeding time (2.2 ± 1.0, 1.9 ± 1.0, 3.2 ± 0.7, and 3.0 ± 0.8 min, respectively) compared with that of the control group (8.2 ± 1.0 min). No significant difference was observed between the PTC-CQDs and HC groups.

### Measurements of coagulation parameters

To determine the hemostatic mechanism of the PTC-CQDs, coagulation parameters (PT, APTT, TT, and FIB) and PLT were evaluated using mouse plasma.

**Fig. 5:**
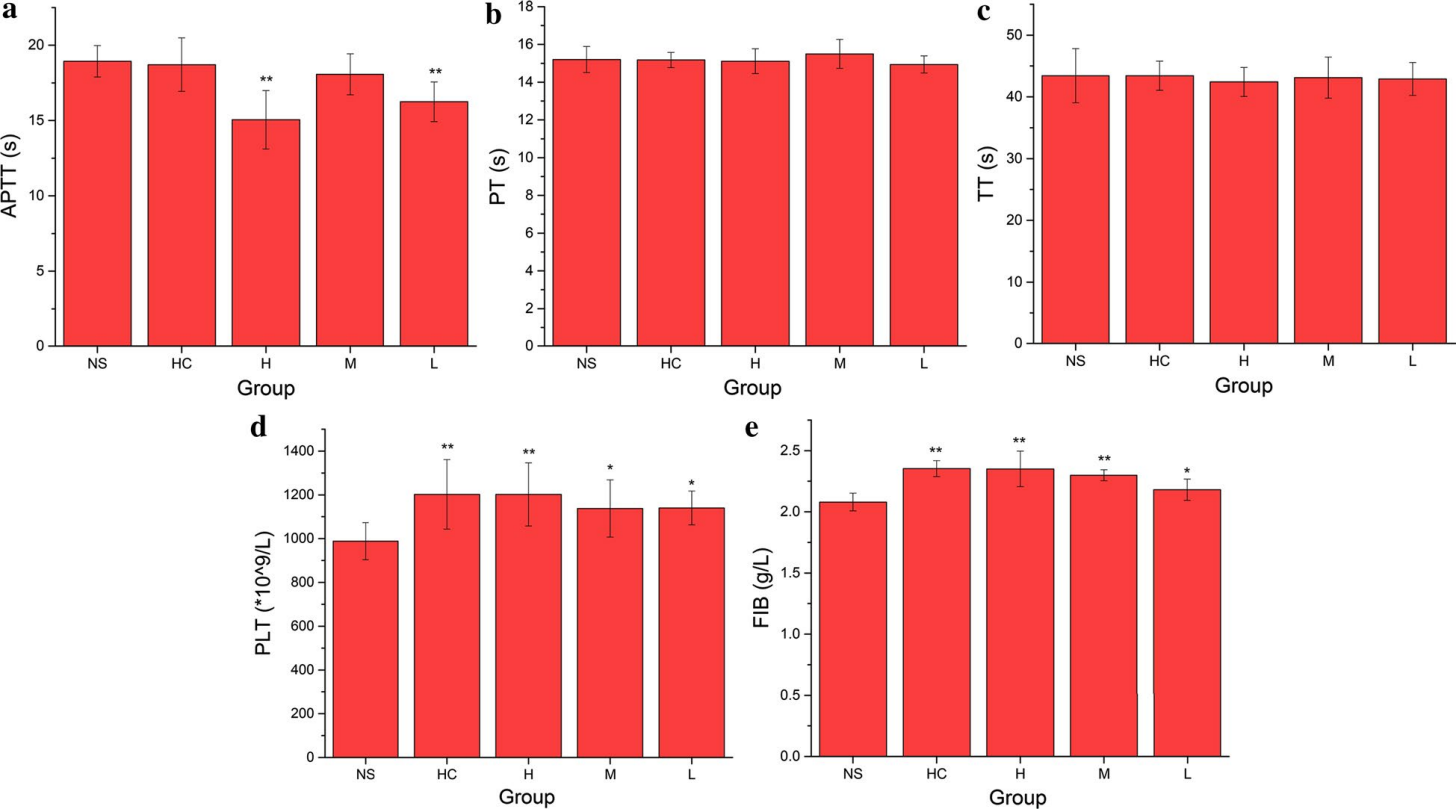
**a** Activated partial thromboplastin time (APTT), **b** prothrombin time (PT), **c** thrombin time (TT), **d** platelet count (PLT), and **e** fibrinogen (FIB) analysis of mice treated with normal saline (NS), hemocoagulase (HC), and different doses of PTC-CQDs (high, H; medium, M; and low, L). **P < 0.01 and *P < 0.05 compared with control group (n = 8)

As shown in Fig. 5, PT and TT values were not significantly different among the four treatment groups. The high (15.05 s) and low (16.25 s) dose of PTC-CQDs decreased APTT significantly (P < 0.01). The PTC-CQDs and HC groups showed a significant increase (P < 0.01) in the FIB (2.35, 2.30, 2.18 and 2.35 g/L, respectively) compared with that of the control group (2.08 g/L). Meanwhile, all doses of PTC-CQDs and HC increased PLT significantly to 1201, 1137, 1140 and 1040 × 10^9^/L, which is in agreement with the results of the bleeding times.

## Discussion

PTC, as well as other charcoal drugs, have a long history of clinical applications for the treatment of hemorrhage diseases in TCM. Modern medical research also indicates the hemostatic function of these charcoal drugs [3, 4]. However, the active constituent(s) are still obscure. It has been reported that carbon dots was obtained from some herbs and plants which is a motivation for us [23]. After found CQDs in aqueous extracts of PTC and being enlightened by the discovery of anti-inflammatory activities of QDs and positive therapeutic actions of QDs on the adverse effects of alcohol overconsumption [8, 9], we turn to study this newly generated CQDs which were absent prior to calcination in this study.

In this study, novel CQDs were discovered and separated from the PTC aqueous extract; they were identified as PTC-CQDs using TEM and HRTEM, as well as FTIR, UV–vis, and fluorescence spectroscopy. Furthermore, to evaluate the component variation and exclude the interference of small molecule organic compounds, a comparative study of the chemical components of PT extraction and PTC-CQDs was carried out by HPLC analysis. As the precursor herb of PTC, PT is mainly composed of flavonoids, such as isorhamnetin-3-*O*-neohesperidoside and typhaneoside [24], and is a widely used herbal medicine in TCM. However, as shown in the HPLC fingerprint results, it is obvious that these small-molecule components and other organics disappeared after calcination.

The results of the pharmacological experiments demonstrate that the obtained PTC-CQDs were highly effective at controlling hemorrhage. To explore the mechanism underlying the hemorrhagic activity of the PTC-CQDs, coagulation parameters were measured in treated SD rats. In our study, APTT decreased, and the FIB and PLT content increased in the PTC-CQDs groups, whereas the PT and TT values showed no significant difference from those of the control group. The blood clotting process includes the endogenous coagulation pathway (intrinsic) and exogenous coagulation pathway (extrinsic). The PT value is correlated with the overall efficiency of the extrinsic clotting pathway, while the APTT is related to the intrinsic coagulation phase. The common coagulation pathway and the activities promoting the transformation of FIB into fibrin in the plasma are related to the TT and FIB levels [25]. An effect on APTT and FIB, but not PT, suggests that the main hemostatic mechanism of PTC-CQDs is associated with the endogenous coagulation pathway (stimulating the intrinsic blood coagulation system and activating the fibrinogen system). This study was a preliminary evaluation of the hemostatic activity and mechanism of the PTC-CQDs, and further investigations are needed to elucidate the deeper underlying mechanisms of these effects. Meanwhile, the discovery of CQDs in PTC and the demonstration of its hemostatic activity in this article provide a new rationale for the material research of PTC and other charcoal drugs.

As appealing substances in modern science, the medical applications of CQDs mainly involve drug delivery and imaging. Because of their high biocompatibility, CQDs have greater potential to sever as therapeutic agents than other types of nanomaterials. However, the reality is that reports of self-bioactivity and related mechanisms of CQDs are uncommon, although there is increasing interest in improving our understanding of the interactions between nanomaterials and living systems. The demonstration of the hemostatic effect of PTC-CQDs and their related mechanism in this article has helped fill the void in this area and has laid a foundation for future drug discovery.

Furthermore, today’s clinical treatment of hemorrhagic disease mainly rely on thrombin. However, it is perishable and unfavorable for long-term retention since it is a kind of protein. Superior to protein, CQDs is more stable and more suitable for longterm preservation, therefore has the potential to become a complementary and alternative therapeutic agent for hemorrhagic conditions under some extreme and inclement circumstances.

## Conclusions

To summarize, a new type of PTC-CQDs was identified and purified from PTC (“a charcoal herb”). The obtained PTC-CQDs were characterized using electron microscopy and spectroscopy. Pharmacodynamic studies revealed a remarkable ability of PTC-CQDs to inhibit hemorrhage in the mouse tail amputation and liver scratch models. Furthermore, these effects may be associated with intrinsic anticoagulation activity and activation of the fibrinogen system, according to the evaluation of the blood coagulation parameters. These results have not only provided a new idea for the material foundation research of charcoal drug, but have also provided new insights into potential biomedical and healthcare applications of CQDs and laid a solid foundation for future drug discovery.

## References

[1] Li F (2011). Effects of carbonized typhae pollen on hemorheological parameters, clotting time and tongue presentations in rats with blood-stasis. Chin J Exp Trad Med Formulae.

[2] Kong XP (2011). Effects of typhae pollen and carbo of typhae pollen on hemorheological parameters and clotting time in blood-stasis rats. Chin J Exp Trad Med Formulae..

[3] Shan MQ (2014). Comparative study on effects of Rubiae Radix et Rhizoma and carbonized Rubiae Radix et Rhizoma on acute blood stasis rat model..

[4] Zhu Y, Qiu Y, Liao L (2015). Evaluation of hemostatic effects of carbonized hair-loaded poly(l-lactic) acid nanofabrics. J Nanosci Nanotechnol.

[5] Drummen GP (2010). Quantum dots-from synthesis to applications in biomedicine and life sciences. Int J Mol Sci.

[6] Qiu J (2015). Fluorescent graphene quantum dots as traceable, pH-sensitive drug delivery systems. Int J Nanomed..

[7] Qiu J (2016). Effects of graphene quantum dots on the self-renewal and differentiation of mesenchymal stem cells. Adv Healthc Mater.

[8] Sun A, Li M, Hu X (2017). Graphene oxide quantum dots as novel nanozymes for alcohol intoxication. Acs Appl Mater Interfaces..

[9] Hu Z (2016). Aqueous synthesized quantum dots interfere with the NF-κB pathway and confer anti-tumor, anti-viral and anti-inflammatory effects. Biomaterials.

[10] Wang K (2013). Systematic safety evaluation on photoluminescent carbon dots. Nanoscale Res Lett.

[11] Li W (2016). Zwitterionic nanogels crosslinked by fluorescent carbon dots for targeted drug delivery and simultaneous bioimaging. Acta Biomater.

[12] Feng T (2016). Charge-convertible carbon dots for imaging-guided drug delivery with enhanced in vivo cancer therapeutic efficiency. ACS Nano.

[13] He G (2016). Rapid solid-phase microwave synthesis of highly photoluminescent nitrogen-doped carbon dots for Fe(3+) detection and cellular bioimaging. Nanotechnology.

[14] Wang J (2015). High performance photoluminescent carbon dots for in vitro and in vivo bioimaging: effect of nitrogen doping ratios. Langmuir.

[15] Jana J (2016). One pot synthesis of intriguing fluorescent carbon dots for sensing and live cell imaging. Talanta.

[16] Wang Y (2016). Multi-doped carbon dots with ratiometric pH sensing properties for monitoring enzyme catalytic reactions. Chem Commun (Camb).

[17] Tao W (2011). Simultaneous determination of eleven major flavonoids in the pollen of Typha angustifolia by HPLC-PDA-MS. Phytochem Anal Pca.

[18] Wu J (2013). Amphiphilic peptide-loaded nanofibrous calcium phosphate microspheres promote hemostasis in vivo. Acta Biomater.

[19] Liu Y (2012). Standardizing a simpler, more sensitive and accurate tail bleeding assay in mice. World J Exp Med.

[20] Van Blerk M (2017). Influence of apixaban on commonly used coagulation assays: results from the Belgian national External Quality Assessment Scheme. Int J Lab Hematol..

[21] Zhang JH (2016). In vivo characterization of hair and skin derived carbon quantum dots with high quantum yield as long-term bioprobes in zebrafish. Sci Rep.

[22] Mewada A (2013). Green synthesis of biocompatible carbon dots using aqueous extract of Trapa bispinosa peel. Mater Sci Eng C Mater Biol Appl.

[23] Chun S (2016). The ethanopharmacological aspect of carbon nanodots in turmeric smoke. Sci Rep.

[24] Cao S (2015). Simultaneous determination of typhaneoside and isorhamnetin-3-*O*-neohesperidoside in rats after oral administration of pollen typhae extract by UPLC-MS/MS. J Chromatogr Sci.

[25] Xin N (2011). Dragon’s Blood extract has antithrombotic properties, affecting platelet aggregation functions and anticoagulation activities. J Ethnopharmacol.

